# Safety risk management for low molecular weight process‐related impurities in monoclonal antibody therapeutics: Categorization, risk assessment, testing strategy, and process development with leveraging clearance potential

**DOI:** 10.1002/btpr.3119

**Published:** 2021-01-06

**Authors:** Haibin Luo, Yuling Li, David Robbins, Sheau‐Chiann Wang, Guoling Xi, Matthew Cox, Simone M. Nicholson, Chenghong Wei, Timothy M. Pabst, William K. Wang

**Affiliations:** ^1^ Purification Process Sciences, Biopharmaceutical Development Department, Biopharmaceuticals R&D AstraZeneca, One Medimmune Way Gaithersburg Maryland USA; ^2^ Analytical Sciences, Biopharmaceutical Development Department, Biopharmaceuticals R&D AstraZeneca, One Medimmune Way Gaithersburg Maryland USA; ^3^ Safety Science, Biopharmaceutical Development Department, Biopharmaceuticals R&D AstraZeneca, One Medimmune Way Gaithersburg Maryland USA; ^4^ Regulatory Affairs, Biopharmaceutical Development Department, Biopharmaceuticals R&D AstraZeneca, One Medimmune Way Gaithersburg Maryland USA

**Keywords:** process clearance, process development, process‐related impurity, risk assessment, safety risk management

## Abstract

Process‐related impurities (PRIs) derived from manufacturing process should be minimized in final drug product. ICH Q3A provides a regulatory road map for PRIs but excludes biologic drugs like monoclonal antibodies (mAbs) that contain biological PRIs (e.g. host cell proteins and DNA) and low molecular weight (LMW) PRIs (e.g., fermentation media components and downstream chemical reagents). Risks from the former PRIs are typically addressed by routine tests to meet regulatory expectations, while a similar routine‐testing strategy is unrealistic and unnecessary for LMW PRIs, and thus a risk‐assessment‐guided testing strategy is often utilized. In this report, we discuss a safety risk management strategy including categorization, risk assessment, testing strategy, and its integrations with other CMC development activities, as well as downstream clearance potentials. The clearance data from 28 mAbs successfully addressed safety concerns but did not fully reveal the process clearance potentials. Therefore, we carried out studies with 13 commonly seen LMW PRIs in a typical downstream process for mAbs. Generally, Protein A chromatography and cation exchange chromatography operating in bind‐and‐elute mode showed excellent clearances with greater than 1,000‐ and 100‐fold clearance, respectively. The diafiltration step had better clearance (greater than 100‐fold) for the positively and neutrally charged LMW PRIs than for the negatively charged or hydrophobic PRIs. We propose that a typical mAb downstream process provides an overall clearance of 5,000‐fold. Additionally, the determined sieving coefficients will facilitate diafiltration process development. This report helps establish effective safety risk management and downstream process design with robust clearance for LMW PRIs.

**Abbreviations**
ICH: International Council for Harmonization of Technical Requirements for Pharmaceuticals for Human Use
LD_50_
: medium lethal dose
LOEL: lowest‐observed‐effect level
NOEL: no‐observed‐effect level
PDE: permitted daily exposure
PRI: process related impurity

## 1 INTRODUCTION

Process‐related impurities (PRIs) are defined in the ICH (International Council for Harmonization of Technical Requirements for Pharmaceuticals for Human Use) Q6B^1^ as “impurities that are derived from the manufacturing process.” PRIs can potentially affect the safety (such as toxicity, immunogenicity, and biological activity) of a drug product.^2^ Regarding toxicity, a statement from Paracelsus is often quoted: “All substances are poisons; there is none which is not a poison. The right dose differentiates a poison and a remedy.”^3^ Therefore, safety risk management to assure residual PRI levels within the safe dose is a must for drug product.^4^ ICH Q3A guidelines provide a regulatory road map and illustrative decision tree for PRIs in chemical drugs,^5^ however it clearly states that biologic drugs are excluded. For biologic drugs, safety risk concerns from PRIs are currently addressed primarily on a case‐by‐case basis and carried out in different ways developed by pharmaceutical companies.^6^

For biologic drugs like mAbs, PRIs generally arise from the cell substrates (e.g., host cell proteins and host cell DNA), the cell culture process (e.g., media components and antifoam), and the purification process (e.g., Protein A leachate from affinity column and detergents used for viral inactivation).^1^ Safety risks of the biologically derived macromolecules or biological PRIs (such as host cell protein, DNA and protein A leachate) are managed through routine testing to assure to be below acceptable ranges.^6^, ^7^, ^8^, ^9^ The risk assessments, process clearance, and assays for biological PRIs have been reviewed in multiple recent publications.^10^, ^11^, ^12^, ^13^, ^14^, ^15^ Putatively acceptable residual levels that are based on human consumption safety history or observations from clinical trials are often used to guide process development, such as 100 parts‐per‐million (ppm) for residual host cell proteins (HCPs)^10^ and 10 ng per dose for DNA.^14^

Most of the upstream PRIs (e.g., vitamins and anti‐foam) and downstream PRIs (e.g. buffers and reagents) have low molecular weight (LMW) compared to the biological PRIs such as HCPs and DNA. These PRIs are usually considered too small to constitute epitopes that can be recognized by the mammalian immune system,^16^ thus the immunogenicity risk is fairly low and can be neglected. Some LMW PRIs (e.g. metal ions) potentially impact protein stability as discussed in a recent review paper^13^ and the impact can be evaluated by stability studies, therefore, these risks are not discussed in this report. We are focused on the safety risk arising from potential toxicity of the LMW PRIs. ICH Q3C (R6),^17^ Q3D (R1),^18^ Q6B,^1^ Q9,^19^ and M7 (R2)^20^ guidelines provide relevant guidance and recommendations, however, safety risk assessment for PRIs in biologics drugs remains complicated.^9^, ^21^, ^22^ Testing every LMW might be the most assuring approach to guarantee no safety risk to patients, but routine tests of all LMW PRIs for every manufacturing lot are unrealistic and unnecessary. Therefore, a science‐based safety risk assessment is highly encouraged to meet regulatory expectations and pharmaceutical companies often implement a safety risk assessment‐guided‐testing strategy for LMW PRIs.^6^, ^22^, ^23^

In this report, we discuss a LMW PRI safety risk management process that consists of multiple stages that can be integrated with CMC development activities. The categorization, risk assessment approaches, testing strategy, downstream clearance, decision tree, and process development aiming for robust PRI removals are also discussed.

## 2 METHOD AND MATERIALS

### 2.1 Chemicals, monoclonal antibodies, column resins, and membranes

The chemicals used in this report were obtained from J.T. Baker (Phillipsburg, NJ, USA) and Sigma‐Aldrich (St. Louis, MO). The mAbs (A and B) are humanized monoclonal antibodies comprising two identical heavy chains and two identical light chains, with molecular weights around 150 kDa. mAb B has slightly higher hydrophobicity (GRAVY index −0.386) than mAb A (GRAVY index −0.414), while mAb A has slightly higher pI (9.3) than mAb B (8.1). Proteins used in this study were purified to greater than 98% monomer purity. MabSelect™ SuRe™ (MSS) Protein A resins were from Cytiva (Marlborough, MA); POROS® HS50™ resins were from ThermoFisher (Waltham, MA). TFF cassettes with Ultracel® 30 kDa membrane were from Millipore (Burlington, MA). Soluble polysaccharide was from BD Bioscience (Franklin Lakes, NJ).

### 2.2 Risk assessment approaches, impurity safety factor and clearance calculation

A risk assessment can be carried out using PDE (permissible daily exposure), which is the maximum acceptable intake per day of an impurity in pharmaceutical products.^17^ A PDE is usually derived preferably from NOEL (no‐observed‐effect level) with the following Equation (1):

(1) $$\mathrm{PDE}=\frac{\text{NOEL}\times \text{Body weight adjustment}}{F1\times F2\times F3\times F4\times F5}$$

where F1 accounts for extrapolation between species, F2 is a factor of 10 to account for variability between individuals, F3 is a variable factor to account for toxicity studies of short‐term exposure, F4 is a factor that may be applied in cases of severe toxicity, and F5 is a variable factor applied if LOEL (low observed effect level) is used.^17^

For a LMW PRI with no available NOEL or LOEL, that is, PDE cannot be determined through Equation (1), a safety risk assessment can be carried out with an impurity safety factor (ISF) calculation.^6^ ISF represents the distance between a toxicity dose and a PRI dose in a product dose. ISF is calculated with the following Equation (2):

(2) $$\text{Impurity safety factor}\;\left(\mathrm{ISF}\right)=\frac{\text{Toxicity dose}}{\mathrm{PRI}\;\text{dose in}\;a\;\text{product dose}}$$

Toxicity dose is the median lethal dose (LD_50_) from animal studies via the relevant administration route. The greater the ISF, the lower the safety risk. The toxicologically acceptable ISF threshold value can be determined carefully based on available data.

PRI dose in a product dose is calculated in the following Equation (3):

(3) $$\mathrm{PRI}\;\text{dose in}\;a\;\text{product dose}=\frac{\mathrm{PRI}\;\text{concentration}}{\text{Product protein concentration}}\times \text{Product dose}$$

For a LMW PRI that has severe toxicity (carcinogenicity and genotoxicity), a risk assessment can be carried out using threshold of toxicological concern (TTC, 1.5 μg/day) for lifelong exposure or acceptable intakes for relevant exposure time recommended in ICH M7.^20^ For a LMW PRI without available toxicity data, this approach can be also used as the most conservative assumption.

Process clearance (fold) for downstream unit operations was calculated with the following Equation (4):

(4) $$\text{Impurity clerance}=\frac{\left(\text{Initial}\;\mathrm{PRI}\;\text{concentration}/\text{Initial protein concentration}\right)\;}{\left(\text{Final}\;\mathrm{PRI}\;\text{concentration}/\text{Final protein concentration}\right)}$$

When the testing result for the PRI was “not detectable,” the assay limit of detection (LOD) was used in the equation.

### 2.3 Chromatography instrument and operations

Chromatographic experiments were carried out on an ÄKTA Avant controlled by Unicorn software version 7.4 (Cytiva, Marlborough, MA). The resin was packed into 0.66 cm inner diameter (ID) Omnifit columns (Diba Industries, Danbury, CT) to a bed height of 19 ± 3 cm. All steps were operated at a flowrate of 300 cm/hour. The purification process was monitored using in‐line ÄKTA sensors (pH, conductivity, and A_280_). The collection of elution product was based on A_280_ collection criterion of 50 mAU for both protein A chromatography and cation exchange chromatography (CEX) experiments.

Protein A chromatography experiments were performed at room temperature under the following conditions: 3 column volume (CV) equilibration buffer (50 mM Tris–HCl, pH 7.4) before loading; loading (30 mg protein/mL resins as load challenge); 3 CV wash buffer (50 mM Tris–HCl, 0.5 M sodium chloride, pH 7.4); 5 CV elution buffer (50 mM sodium acetate pH 3.6); 3 CV strip buffer (100 mM acetic acid) for column regeneration; 3 CV sanitization buffer (0.1 M sodium hydroxide); and 3 CV storage buffer (20% ethanol) for column storage. LMW PRIs were spiked into the feed at desired concentrations. The flow through and wash fractions were collected from 0.75 CV post‐load or wash start to 1.25 CV post‐load or wash end, respectively. The two mAbs were prepared separately at 5 mg/ml with the equilibration buffer as feed. The feeds were spiked with the selected LMW PRIs to the targeted concentrations.

CEX chromatography experiments were operated at room temperature under the following conditions: 3 CV equilibration buffer (50 mM sodium acetate, pH 5.0) for pre‐loading equilibration; loading (30 mg/mL resin as load challenge); 3 CV wash buffer (50 mM sodium acetate, pH 5.0); 5 CV elution buffer (50 mM sodium acetate, 500 mM sodium chloride, pH 5.0); 3 CV strip buffer (50 mM Tris–HCl, 0.5 M sodium chloride, pH 7.4); 3 CV sanitation buffer (1 M sodium hydroxide); 3 CV storage buffer (0.1 M sodium hydroxide). The flow through fraction was collected from 0.75 CV after load start to 1.25 CV after wash end. The two mAbs were prepared separately at 5 mg/ml with the equilibration buffer as feeds. The feeds were spiked with LMW PRIs to the targeted concentrations.

### 2.4 Tangential flow filtration (TFF) experiments and clearance data analysis

Tangential flow filtration experiments were carried out using Pellicon XL Cassettes from Millipore (Burlington, MA). TFF experiments were operated under the following conditions at room temperature: the membrane was flushed with equilibration buffer (50 mM sodium acetate, 0.2 M sodium chloride, pH 5.0); loading; ultra‐concentration to a target concentration of 50 or 100 mg/ml; 6 diavolume (DV) diafiltration with the final formulation buffer (25 mM Histidine, pH 6.0) and diafiltration using a constant retentate volume; chase with formulation buffer; 0.5 M sodium hydroxide for cleaning; and 0.1 M sodium hydroxide for membrane storage. The impurity to be tested was spiked before the start of diafiltration. Samples were taken after each DV and tested by the corresponding qualified assay. Clearance of the tested PRIs was analyzed with the following Equation (5)^24^:

(5) $$C=C_{0}\times e^{\left(-\mathrm{NS}\right)}$$

where *C* is the final concentration of the PRI, *C*_0_ is the initial PRI concentration, *N* is the number of DV, and *S* is the sieving coefficient. *S* was determined by fitting the equation to the experimental data. Unless mentioned otherwise the fittings had *R* factors greater than 95%.

### 2.5 Analytical assays and sample testing

The assays for the 13 LMW PRIs evaluated were qualified for precision and recovery. The amount of an LMW PRI in each test sample was determined based on the corresponding calibration curve generated with the standards. Beta‐mercaptoethanol (BME) and monothioglycerol were measured with a fluorescence spectroscopy assay that uses a fluorometric thiol reagent to generate fluorescent adduct upon reacting with BME. Caprolactam was measured using the assay described by Bradly et al^25^ with minor modifications. Dextran sulfate was measured by mixing sample with 1,9‐dimethyl‐methylene blue dye. The blue dye binds to dextran sulfate to form a complex that absorbs at 530 nm. Ethylenediaminetetraacetic acid (EDTA) and Pluronic F68 were measured by an assay based on anion exchange column separation with diode‐array detection at 254 nm. The soluble polysaccharide was measured by a commercial kit from ThermoFisher (Waltham, MA). Methionine sulfoximine (MSX) was measured by an assay based on reverse phase high performance liquid chromatography and monitored with fluorescence detection (excitation at 230 nm and emission at 450 nm). PEG 8000 was measured by an assay based on reverse phase high performance liquid chromatography and evaporative light scattering detection. The measurements of simethicone and copper ion were based on silicon and copper level, respectively, that were detected by inductively coupled plasma mass spectrometry. Triton X‐100 was measured by an assay based on reverse phase high performance liquid chromatography and ultraviolet absorbance at 225 nm. Tropolone was measured by an assay based on ultraviolet spectrophotometry and hallmark light absorbance at 392 nm.

## 3 RESULTS AND DISCUSSION

### 3.1 Safety risk management for LMW PRIs

**FIGURE 1:**
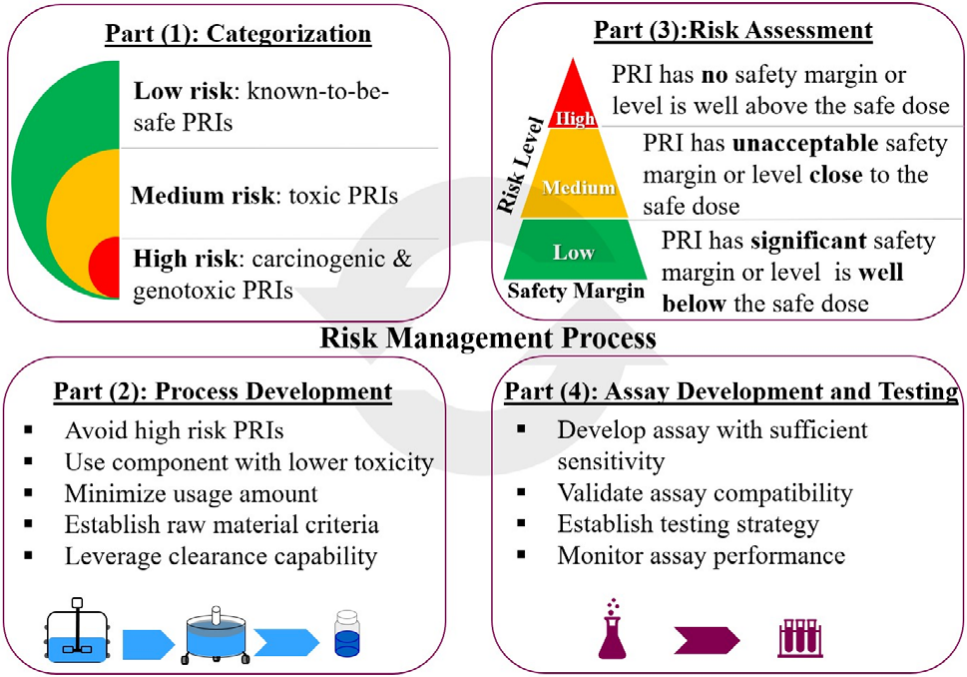
Schematic of safety risk management process for LMW PRIs

Safety risk management for LMW PRIs consists of four interactive parts (Figure 1). Part (1) is the categorization of LMW PRIs using an initial safety risk identification step based the preliminary manufacturing process. LMW PRIs are categorized on the basis of their safety risk (or toxicity) levels, that is, low, medium, and high. The safety risk level is determined according to the safety and toxicity data available in scientific literature and public databases, as well as information from regulatory guidance.^4^ In brief, PRIs carry low safety risk are considered as “known‐to‐be‐safe” and can be eliminated from the safety risk management process. PRIs with reported medium toxicity are considered to pose medium risk, while PRIs with reported genotoxicity and carcinogenicity are considered to pose high risk. PRIs with medium and high risks are carefully managed in the following three parts. The Part (1) categorization mainly focuses on toxicity of PRI and the risk associated with usage amount is evaluated in the following Part (3). Part (2) consists of process development. As a rule of thumb, high‐risk PRIs should be avoided; medium‐risk PRI usage should balance risk and process benefit after process clearance knowledge is obtained; while low‐risk PRI usage may have more flexibility to maximize process benefits. Additionally, acceptance criterion can be set up for raw materials to simplify risk management and reduce the testing burden. Maintaining process clearance data for LMW PRIs builds the knowledge base about the process clearance potential for different PRIs, which has the potential to reduce future process development activities. Part (3) is a safety risk assessment for the remaining high‐ and medium‐risk PRIs to further define their safety risk levels. Generally, when a PRI has a significant safety margin or its residual level is well below the safety dose, the PRI can be considered to pose a low safety risk. PRIs with limited safety margin (a level close to the safety dose) or no safety margin (the level is at or above the safety dose), additional actions must be taken (such as testing or process change) to minimize their safety risks. Part (4) consists of assay development and testing for LMW PRIs that are identified in Part (3) to demonstrate process clearance. A suitable assay with sufficient sensitivity needs to be developed and confirmed compatible with the samples to be tested. Appropriate testing points need to be selected and a PRI testing plan is established for GMP manufacturing.

Overall, implementation of safety risk management processes help to systematically eliminate safety risk and meet regulatory expectations, as well as streamline CMC development.

### 3.2 Categorization, safety risk assessment approaches, and decision tree for LWM PRIs

**FIGURE 2:**
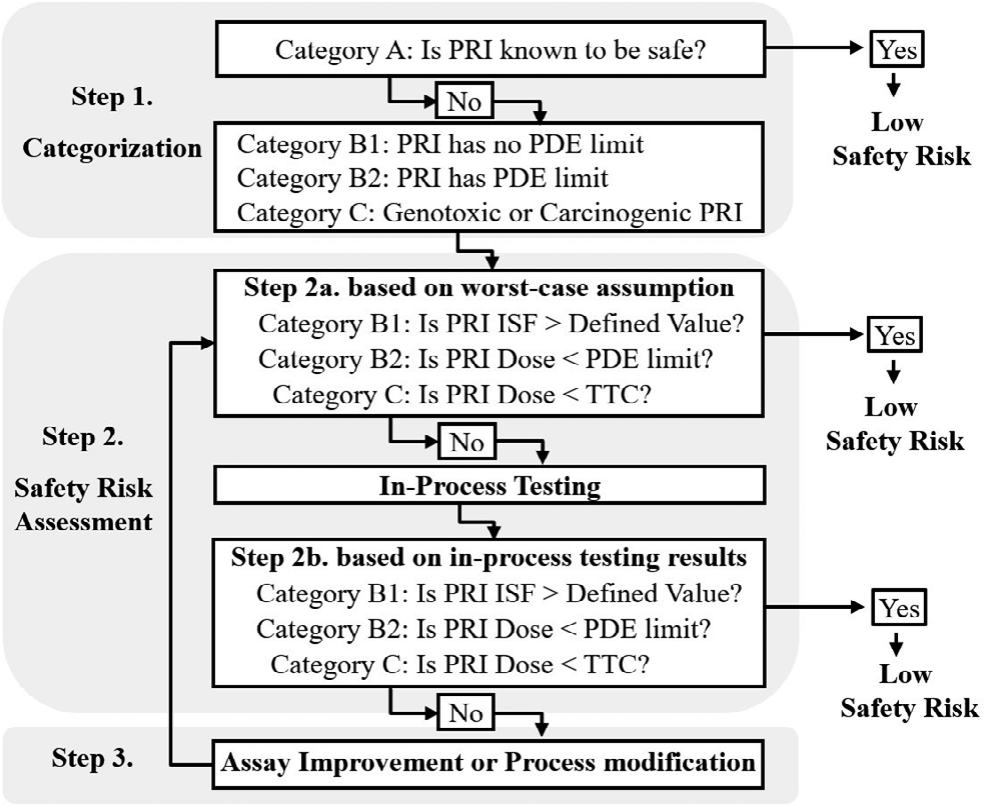
Decision tree for LMW PRIs risk assessment. PDE, permitted daily exposure; TTC, threshold of toxicological concern

Categorization of LMW PRI is an initial risk identification step. Generally, LMW PRIs can be categorized into three groups based on toxicological risks: A, B, and C (Figure 2).

Category A contains LMW PRIs that inherently pose no safety risk and are termed “known‐to‐be‐safe” PRIs within the safety risk assessment. Many LMW PRIs derived from upstream processes are nutrients (such as amino acids, vitamins, salts, lipids, carbohydrates and trace elements) required for cell growth. Many of these PRIs can be found in humans as naturally existing chemicals, that is, human metabolites. Metabolites and the concentration range in humans can be found in the Human Metabolome Database.^26^, ^27^ ICH Q3A^5^ guideline states that “…impurities that are also significant metabolites present in animal and/or human studies are generally considered qualified” to be in the drug substance. Weidolf et al proposed an utilization of metabolite exposure and maximum theoretical impurity concentration to define qualification of PRI in drug product.^28^ With usually low concentrations being used, the metabolite type LMW PRIs should pose no safety risks and can be considered to be “known‐to‐be‐safe.” For downstream processes, most buffers and salts (such as sodium acetate and sodium chloride) are in the GRAS (Generally Recognized as Safe) list issued by the U.S. FDA. Some PRIs (polysorbate 80 and arginine) are excipients in approved drugs and have been proven safe. Detailed risk assessments and in‐process testing are usually unnecessary for Category A PRIs and can be eliminated from the rest workflow as showed in Figure 2.

On the contrary, PRIs with reported acute severe toxicity (such as genotoxicity and carcinogenicity) have the highest safety risks and thus are classified as Category C PRI (such as nitrosamines^20^, ^29^). Category C PRIs should be avoided as a rule of thumb. LMW PRIs with toxicological risks between Categories A and C, that is, medium safety risks, are classified into Category B. As shown in Figure 2, Category A is considered to pose no safety risk, while Categories B and C PRIs need to follow Step 2 risk assessment for further evaluation.

Generally, safety risk assessment involves comparing the PRI dose level in a single dose of the product to the established safety dose level. Depending on the PRI category, different approaches can be used for risk assessment. For Category B PRIs that have established PDE limit (Category B2), risk assessment is carried out by comparing the PRI dose in a product dose to the PDE. The PRI dose in a product dose is calculated using Equation (4). PDE values for common PRIs in chemical drugs and elemental impurities are recommended in ICH Q3C^17^ and Q3D,^18^ respectively. Unfortunately, many of the LMW PRIs used in biologic manufacturing processes are not provided with PDE values in these two guidelines. For PRIs that do not have PDE values available, ICH Q3C recommends an equation (Equation (3)) to generate PDE from NOEL or LOEL.^17^ However, for some PRIs utilized in biologic drugs, NOEL or LOEL values are not available.^23^ Schenerman et al^23^ proposed an approach termed “impurity safety factor (ISF)” to measure the distance between the PRI level in a dose of product to the established toxicity dose. The PRI is considered to pose no safety risk only when the ISF is greater than the defined threshold value. Subsequently, the CMC Biotech Working Group, consisting of industry experts, adopted this ISF approach in a white paper entitled “A Mab: A Case Study in Bioprocess Development”^6^; and the PhRMA working group included the ISF approach in its advice on applying “quality by design for biotechnology products.”^7^ To measure safety risk of Category B1 PRIs, ISF values can be calculated using Equation (2). The threshold ISF value can be carefully determined based on the dose–response relationship.^23^ For Category C PRIs, risk assessment is performed by comparing PRI dose in a product dose to a TTC value (typically 1.5 μg/day).^20^

Step 2 safety risk assessment is divided into two sub‐steps, as shown in Figure 2. Step 2a risk assessment uses worst‐case assumptions. The main assumption is that the PRIs are copurified with the product to final drug substance, that is, process clearance is not considered. If the resultant ISF is greater than the defined threshold value or the worst‐case PRI level is less than the established safety dose (PDE and TTC), the PRI can be considered a low safety risk. Otherwise, process clearance needs to be demonstrated for this PRI by analytical testing. Step 2b safety risk assessment is performed based on the in‐process testing results. Similarly, if the resultant ISF value determined using testing results is less than the defined threshold value or the residual PRI dose is lower than PDE and TTC, the PRI can be considered as a low safety risk. If the resultant ISF value is too high then either the process (e.g. reduce the amount of PRIs used or improve the process to get sufficient removal) or the analytical method (e.g., poor sensitivity) need to be improved, and the ISF should be recalculated until it is acceptable.

Additionally, for PRI that has no available safety/toxicity data or PRI without chemical identity, risk assessment can be carried out assuming that the PRI has the highest safety risk and follow the assessment workflow for a Category C PRI.

### 3.3 Safety risk assessment of LMW PRIs in a mAb

**FIGURE 3:**
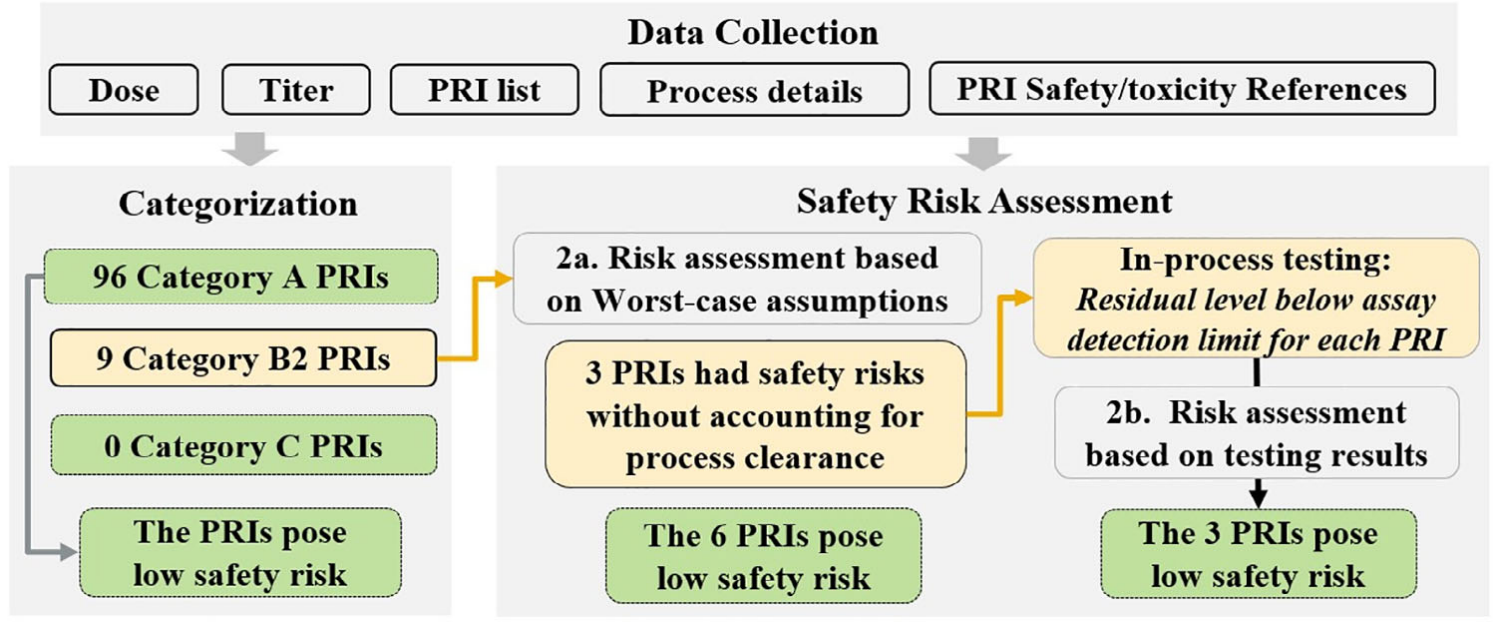
An example for safety risk assessment for LMW PRIs

Figure 3 illustrates the safety risk assessment progress for a mAb. The process started by collecting data including the projected maximum dose of the mAbs, cell culture titer ranges, process (upstream and downstream) details, and safety/toxicity references. With the data input, 105 LMW PRIs were categorized as shown in Figure 3: 96 Category A PRIs were identified and there were no Category C PRIs. These 96 PRIs were considered to pose no safety risk and were eliminated from the assessment workflow. The remaining nine PRIs (after eliminating the 96 PRIs) were identified as Category B2 PRIs and therefore Step 2a assessment was carried out for the nine PRIs. Step 2a results suggested that six out of the nine PRIs posed no safety risk even without accounting for process clearance. The remaining three PRIs were considered to pose safety risks without accounting for process clearance. Accordingly, in‐process testing for three PRIs was added to the testing plan for GMP manufacturing, and testing was carried out accordingly at the viral filtration step to demonstrate the process clearance. The three PRIs (an antifoam, an anti‐shear protectant, and a chemical reagent for cell line selection) were not detected in the samples by the corresponding assays. Step 2b assessment was carried out using the corresponding assay detection limits and the results demonstrated that the three PRIs posed no safety risks. Therefore, the 105 PRIs in this example posed no safety risk and their safety risks were successfully managed.

### 3.4 Integration of safety risk management progress with CMC development activities

**FIGURE 4:**
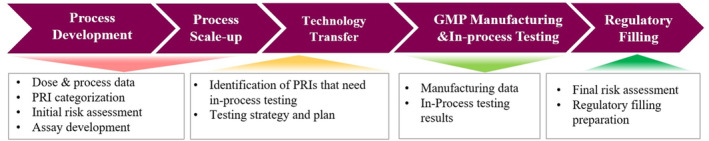
Interactions of LMW PRI risk management with CMC development activities

Figure 4 illustrates the alignment of the safety risk management process with CMC development activities. The safety risk management process can begin once a preliminary manufacturing process is determined. PRI related process information (PRI list, usage concentration, process flow, and PRI introduction point) and the projected product dose are used for the initial safety risk assessment. The initial PRI safety risk assessment typically uses data from bench scale and serves a first round of risk identification, and assays should be developed as needed. When data from process scale‐up runs become available, the risk assessment is reperformed to confirm the initial safety risk. With the assessment results, a testing strategy and testing plan for GMP manufacturing can be established. After the GMP manufacturing is completed, testing data are collected and used for a final step of the safety risk assessment. Similar safety risk assessments can be performed for every GMP manufacturing and the results are tracked. This data not only can prove the robust removal of LMW RPIs but facilitates the commercial manufacturing process control strategy .

### 3.5 Clearance data for 6 LMW PRIs from 28 mAbs

**FIGURE 5:**
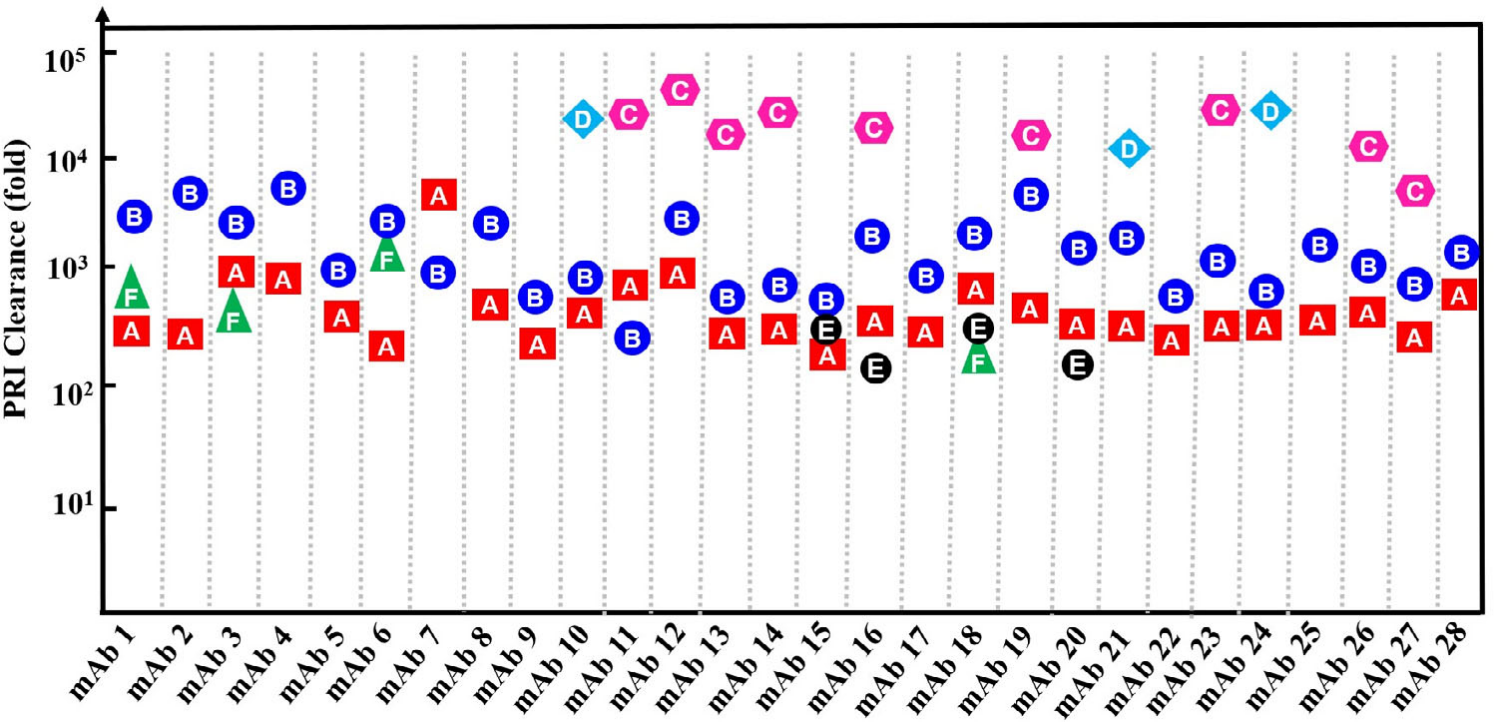
Process clearance of LMW PRIs by downstream process for 28 mAbs. A, B, C, D, E, and F represent six different LMW PRIs. The impurity clearance (fold) was calculated using by Equation (4). The *Y*‐axis in log‐10 scale represents the impurity clearance in fold. The 28 mAbs have similar molecular weights of 145–152 kDa. mAb 1, 9, 16, 19, and 24 have isoelectric point (pI) at 6.5–7.0; and mAb 2, 3, 11, 22 have p*I* at 7.0–8.0; the rest mAbs have pI at 8.0–8.5. mAb 1 and 3 are IgG4; mAb 8 and 9 are IgG2; and the rest mAbs are IgG1. The data for mAb 7, 11, 12, 13, 20, 22, 24, and 77 is from 500 L scale; the rest is from 2,000 L scale

Removal of LMW PRIs by downstream manufacturing processes is one aspect that determines their risk level. Therefore, downstream process clearance is critical for the PRI safety risk mitigation. Knowing the process clearance potential should help risk mitigation. Figure 5 shows the clearance data from large‐scale GMP manufacture of 28 different mAbs. A, B, C, D, E, and F represent six different PRIs that need in‐process testing based on Step 2a safety risk assessment results. The 28 mAbs were manufactured with similar downstream platform processes (including the same process flow, the same chromatography resins, and highly similar operation concentrations). The testing point for all six PRIs was at the viral filtration step rather than the final tangential flow filtration (TFF) step to reduce the impacts of high protein concentration of TFF product to the impurity assays. The testing results were all “not detectable” as measured by the corresponding assays. The PRIs in the 28 mAbs posed no safety risks after the risk assessment with the testing results. The clearance values were calculated with Equation (4). The usage concentration was different for different PRIs in the same mAb, while the usage concentration for the same PRI in different mAb was also different. As expected, a broad range of clearances for different PRIs was observed. For example, in mAb 1, PRI A, B, and F had 220‐, 521‐, and 2,233‐fold clearance, respectively. PRI A had 220‐fold clearance in mAb 1 and significantly greater clearance of 3,544‐fold in mAb 7. PRI C and D generally had greater clearance than the PRI A, B, E, and F. The maximum demonstrated clearance for the studied PRIs was greater than 10,000‐fold and the minimum clearance was greater than 100‐fold. All six of the PRIs were effectively removed and the residual levels determined by the in‐process testing results were acceptable for toxicology considerations and thus posed no safety risks. These results suggest the downstream process used by the 28 mAbs robustly removed the six different PRIs even when some PRIs were used at significantly higher concentrations. For the same LMW PRI, removal by the downstream process varied, but was generally similar across different mAbs. Additionally, the potential clearance can be greater as the demonstrated clearance is limited by assay detection limit. The demonstrated clearance is the sum of multiple steps, therefore the clearance of each step is unclear. Understanding the maximum clearance of each step is helpful for process design (such as PRI usage concentration and where to introduce) as well as selecting the appropriate testing point. Additionally, it is possible that clearance potential of the same purification step can vary for different PRIs, which can be caused by undesired retention mechanisms. These retention mechanisms can be weak interactions between resin or mAb and PRIs due to certain physical properties, such as electrostatic attractions and hydrophobic interactions. Therefore, it is important to evaluate mAb downstream processes, with PRIs possessing different physical properties.

### 3.6 Typical downstream process for mAbs and the clearance potentials for LMW PRIs

**FIGURE 6:**
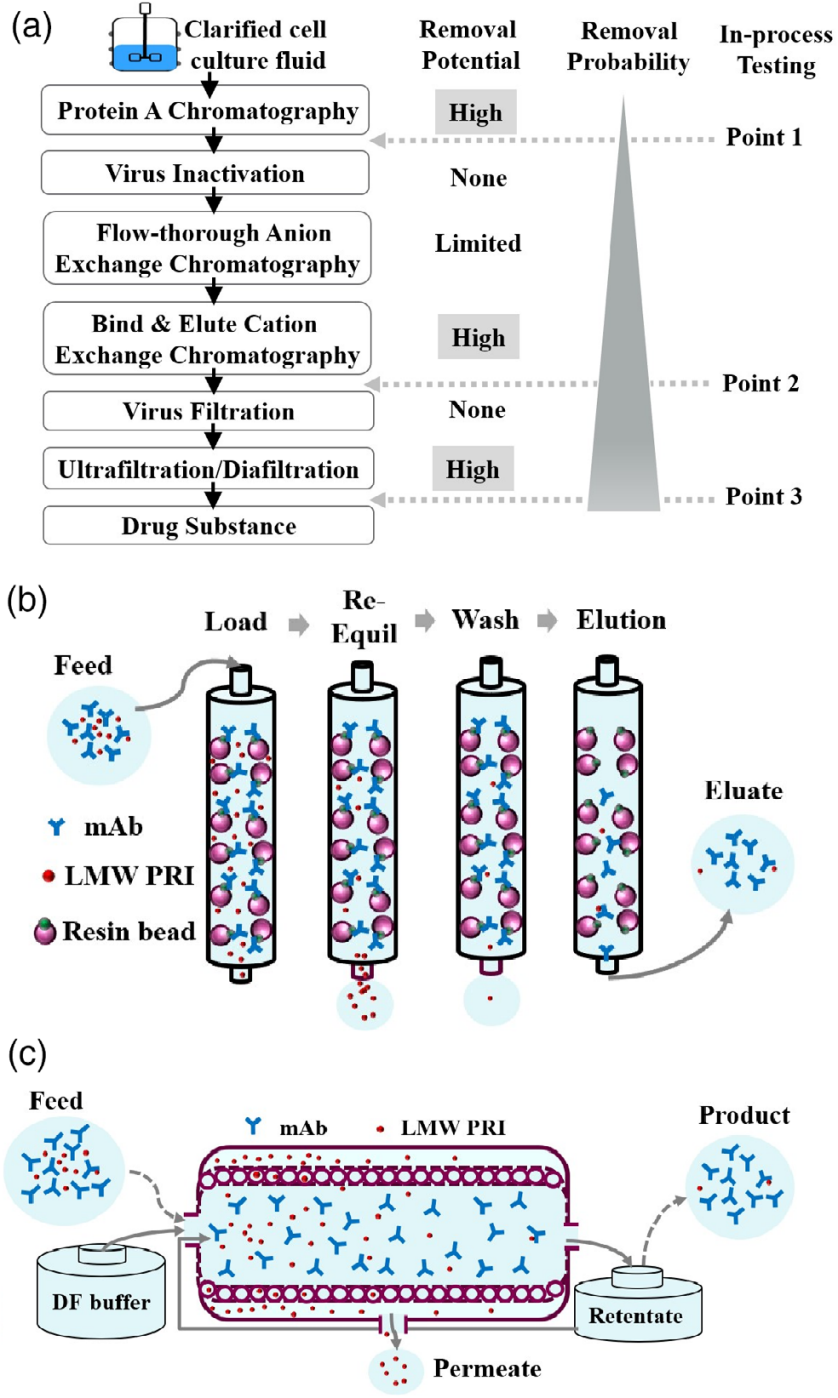
Downstream process flow and clearance potential. (a) A typical downstream process for mAbs. (b) Schematic illustration of LMW PRI removal in Protein A chromatography and cation exchange chromatography. (c) Schematic illustration of LMW PRI removals during diafiltration process

Figure 6a shows a typical downstream process for mAbs, similar to the processes discussed in a recent review article.^30^ Protein A chromatography, cation exchange chromatography operated in bind‐and‐elute mode and a diafiltration step should have significant clearance potential for LMW PRIs, while virus inactivation and virus filtration are not expected to contribute to clearance. Figure 6b demonstrates how PRIs are removed in Protein A chromatography and cation exchange chromatography. Briefly, mAbs are retained on the column through bindings to the resin ligands and LMW PRIs are removed by flowing through the column; any residual PRIs can be further removed by the following re‐equilibration and/or wash steps. Most LMW PRIs in the feed are removed and should be absent in the eluate. Figure 6c illustrates how LMW PRI can be removed during diafiltration. Diafiltration of tangential flow filtration (TFF) is used to prepare the mAb of interest into the target formulation. The typical pore size (or molecular weight cut‐off) of a TFF membrane used for mAbs (150 kDa) ranges from 30 to 50 kDa, therefore, mAbs are effectively retained and can be recovered in “retentate.” Due to the significantly smaller size, PRIs can pass freely through TFF membranes and are removed in the “permeate.” The removal of PRI depends on the number of diafiltration cycles, that is, diavolume (DV); the more diafiltration cycles, the greater the removal for LMW PRIs. Typically, greater than five DVs diafiltration is used to achieve a fair balance between effective buffer exchange and consumption of diafiltration buffers.^30^

To fully evaluate the clearance potential of downstream processes, we carried out systemic spiking and clearance studies on three downstream unit operations (Protein A chromatography, cation exchange chromatography, and tangential flow filtration) using 13 PRIs that have been reported to be commonly used in manufacturing processes (summarized in Table 1). These PRIs have molecular weights ranging from 70 to 9,000 g/mol and have different physical properties such as charge and hydrophobicity.

**TABLE 1:** Summary of the LMW PRIs used in the clearance studies

LMW PRIs	Usage in process	MW (g/mol)	Physical properties	Usage purpose
BME	Upstream	78.1	Uncharged	Support cell growth^36^
Copper ion	Upstream	79.5	Positively charged	Support cell growth and facilitate antibody disulfide bond formation^37^
Caprolactam	Upstream; downstream	113.2	Uncharged and hydrophobic	Leachate from containers and tubes^38^
Dextran sulfate	Upstream	~4000	Negatively charged	Prevent cell aggregation^39^
EDTA	Downstream	292	Negatively charged	Prevent enzyme inhibition and disulfide bond reduction^40^
Polysaccharide	Upstream	~5000	Uncharged	Support cell growth^41^
MTG	Upstream	108.1	Uncharged	Support cell growth^42^
MSX	Cell banking	180.2	Positively charged	Support cell selection^43^
PEG 8000	Upstream; downstream	~8000	Uncharged	Protein stabilizer^44^
Pluronic F68	Upstream; downstream	~8000	Uncharged surfactant	Cell shear protectant and protein stabilizer^45,46^
Simethicone	Upstream	238.5	Uncharged	Antifoam^47^
Triton X‐100	Downstream	625	Nonionic surfactant	Virus inactivation^48^
Tropolone	Upstream	122.1	hydrophobic	Support cell growth^49^

Abbreviations: BME, beta‐mercaptoethanol; EDTA, ethylenediaminetetraacetic acid; MTG, monothioglycerol; MSX, methionine sulfoximine.

### 3.7 Clearance of LMW PRIs in Protein A chromatography

**FIGURE 7:**
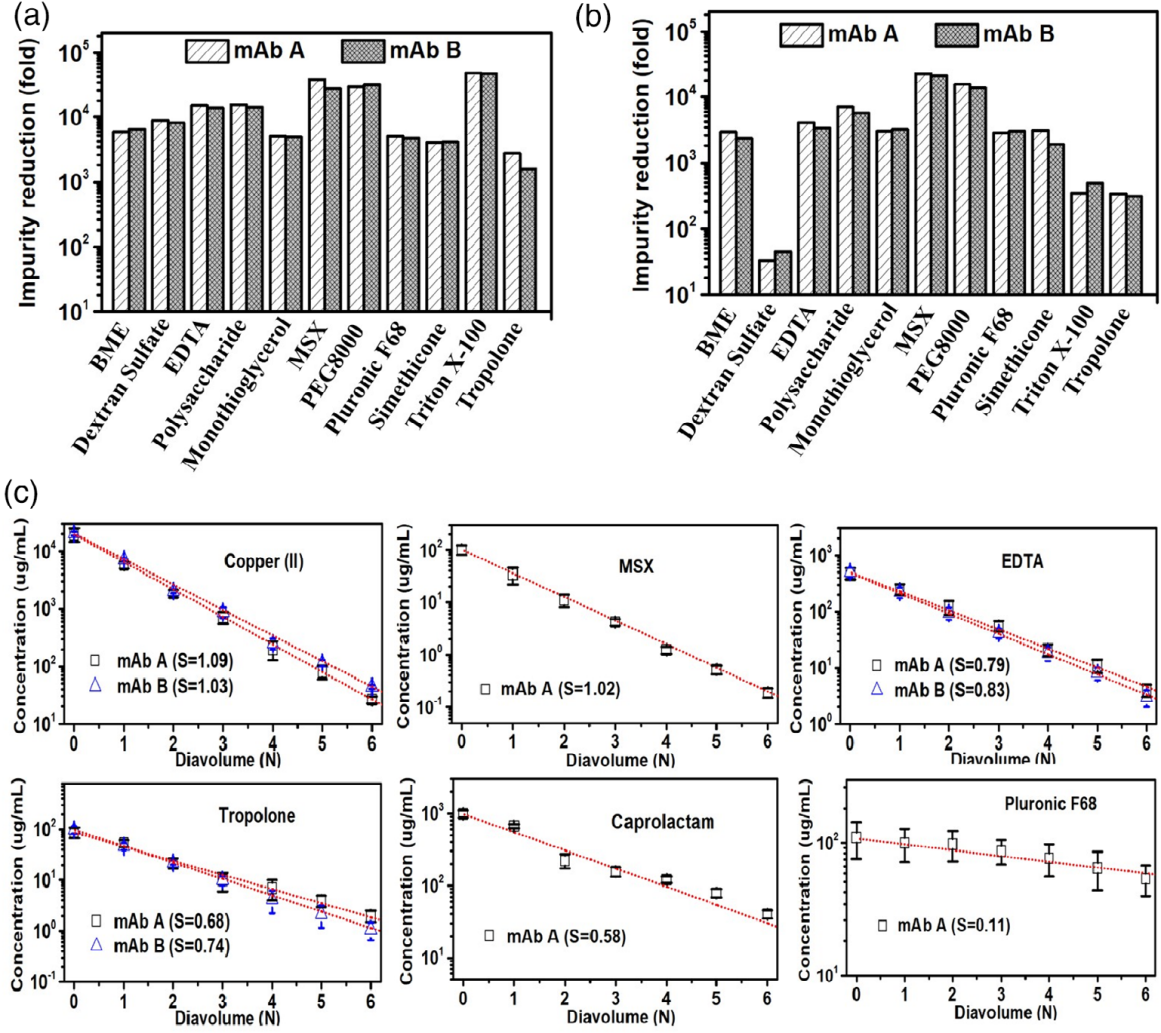
Downstream clearance for LMW PRIs. The clearance obtained in (a) Protein A chromatography, (b) cation exchange chromatography (c) and during diafiltration. In (a) and (b), protein concentration in the load was 5 mg/ml. In (c), protein concentration was 100 mg/ml during diafiltration and the dotted line is a fit of the data using Equation (5) with a sieving coefficient, *S*

The results from the spiking/clearance study on Protein A chromatography are summarized in Table 2 and Figure 7a. As shown in Table 2, the spiked PRIs were mainly in the flow‐through fractions. Generally, these PRIs were not retained by the Protein A column. The residual levels of all studied PRIs in the eluate fraction were very low, compared to their starting levels in the feed. The results suggest that Protein A chromatography provides a robust clearance for LMW PRIs, consistent with the recent study on different PRIs.^4^

**TABLE 2:** Clearance of LMW PRIs during Protein A chromatography

LMW PRIs	Concentration in feed (μg/ml)	Concentration in flow‐through (μg/ml)	Concentration in wash (μg/ml)	Concentration in eluate (μg/ml)
BME	1,005	931	<1	<1
Dextran sulfate	977	691	3.3	<0.5
EDTA	1,633	1,319	<0.5	<0.5
Polysaccharide	0.47	0.40	<0.00005	0.0001
MTG	648	623	<0.8	<0.8
MSX	81	76	<0.01	<0.01
PEG8000	16,726	15,452	<15	<15
Pluronic F68	33,285	29,111	<2.5	<5
Simethicone	240	220	<1	<1
Triton X‐100	10,975	9,544	1.3	<0.1
Tropolone	1,938	1,681	<1	2.8

Interestingly, low levels of dextran sulfate and Triton X‐100 were detected in the Wash fractions but they were not detected in the Eluate fraction. The results indicate that these two PRIs were weekly retained on the Protein A column during loading, likely due to weak interactions with the mAb proteins or the resins. These weak interactions were effectively disrupted by the wash condition because the two PRIs were not detected in the Eluate. Therefore, a wash condition can further improve PRI removal capability of Protein A chromatography. Due to potential weak interactions between LMW PRIs and the mAb, mAb properties (such as charge and hydrophobicity) may affect LMW PRIs removal. As shown in Figure 7a, clearance of the same PRI was similar between the two different mAbs, suggesting the contribution from mAbs to PRI removal may be negligible.

As shown in Figure 7a, more than 1,000‐fold clearance was achieved for all tested LMW PRIs. Clearance for EDTA, polysaccharide, MSX, PEG8000, and Triton X‐100 were greater than 10,000‐fold. Along with the historical data summarized in Figure 5 and the recent publication,^4^ we propose that similar clearance for the same LMW PRI can be achieved for different mAbs in Protein A chromatography. These results suggest that Protein A chromatography provides robust clearance for LMW PRIs. Furthermore, certain LMW PRIs, like dextran sulfate and Triton X‐100 in our study, may be retained on the Protein A column by weak interactions; however, a wash step prior to the elution can efficiently remove these PRIs by effectively disrupting the weak interactions.

### 3.8 Clearance of LMW PRIs in cation exchange chromatography

The results from spiking/clearance studies from cation exchange chromatography are summarized in Table 3 and Figure 7b. Except for dextran sulfate and Triton X‐100, all of the spiked PRIs in the feed were removed in the column flow‐through, and the remaining level in the elution fraction was very low with most as “not detectable.” The clearance for BME, polysaccharide, monothiol glycerol, Pluronic F68, and simethicone was more than 1000‐fold. Unlike Protein A chromatography, removal of dextran sulfate by cation exchange chromatography was not as effective compared to the other tested PRIs. Considering that dextran sulfate is negatively charged under the pH conditions,^31^ its interactions with the positively charged mAbs may reduce the clearance. The removal of Triton X‐100 on cation exchange chromatography was also less than that on Protein A chromatography. Similar clearance of dextran sulfate and Triton X‐100 on cation exchange chromatography were also observed on mAb B (Figure 7(b)), suggesting that mAb‐specific interactions are unlikely the major reason. The retention mechanism is likely weak interactions between Triton X‐100 and the mAb; however, further investigation is needed to confirm this hypothesis.

**TABLE 3:** Clearance of LMW PRIs during cation exchange chromatography

LMW PRIs	Concentration in load (μg/ml)	Concentration in flow‐through (μg/ml)	Concentration in eluate (μg/ml)
BME	869	651	<1
Dextran sulfate	643	330	45.6
EDTA	615	508	<0.5
Polysaccharide	0.47	0.45	0.0006
MTG	661	520	<0.8
MSX	68	56	<0.01
PEG8000	16,797	12,539	<20
Simethicone	240	220	<1.3
Triton X‐100	9,885	5,622	94.2
Tropolone	312	211	<1.8

### 3.9 Clearance of LMW PRIs during diafiltration

For diafiltration, we carried out spiking studies with six PRIs representing several types of PRIs with different chemical properties including: copper ion (positively charged), MSX (charged but neutral), EDTA (negatively charged), tropolone (slightly hydrophobic), caprolactam (highly hydrophobic), and Pluronic F68 (surfactant). The 30 kDa MWCO (molecular weight cut‐off) TFF membrane effectively retained 150 kDa mAb proteins while most of the PRIs passed through the TFF membrane during diafiltration into the permeate. The concentration as a function of diavolumes (DVs) for the PRIs tested is shown in Figure 7c. As shown in Figure 7c, copper ion and MSX were effectively removed by diafiltration. A typical 6 DV diafiltration resulted in greater than 300‐fold clearance for these two PRIs. Equation (5) was fitted to the data shown in Figure 7c and the sieving coefficients for copper(II) and MSX were estimated to be 1.09 and 1.02, respectively, suggesting nearly ideal sieving. Significant removal of EDTA, tropolone, and caprolactam was also achieved by diafiltration, although the clearance for the three PRIs was not as effective as copper ion and MSX. Accordingly, the obtained sieving coefficients for these three PRIs were in the range of 0.58–0.83. Very limited clearance was obtained for Pluronic F68, even with a spiked concentration (450 μg/ml) that was significantly lower than the critical micelle concentration (1,900 μg/ml).^32^ Poor clearance is expected when the Pluronic F68 concentration is higher than critical micelle concentration because the size of the micelles is greater than the TFF membrane MWCO. The sieving coefficient for Pluronic F68 was estimated out to be 0.11.

Copper ion had a sieving factor slightly greater than one likely due to electrostatic repulsions between the positively charged copper ion and the positively charged mAb.^33^, ^34^ Similarly, EDTA removal was not as effective as copper ion or the neutrally charged MSX (possessing one negatively and one positively charged group) due to electrostatic attractions with the positively charged mAb. As a result of the interaction with the mAb, extended diafiltration would be needed to achieve greater clearance of the PRI. For example, to achieve 100‐fold clearance for EDTA, it would take six DVs. Removal of tropolone and caprolactam was compromised with sieving coefficients of 0.7 and 0.6, respectively. Tropolone and caprolactam are hydrophobic and their clearance is likely associated with weak interactions with the mAb. Therefore, for negatively charged or hydrophobic PRIs, mAb properties, such as charge and hydrophobicity, are expected to affect the clearance of these PRIs. To test the hypothesis, diafiltration clearance studies for copper ion, EDTA, and Tropolone were performed on mAb B. Under the diafiltration buffer pH of 6.0, in theory, mAb A (pI 9.3) likely carries more positive charges than mAb B (pI 8.1) while mAb B is more hydrophobic than mAb A. Clearances for copper(II), EDTA, and tropolone were similar for the two mAbs. The results suggest that different mAbs have negligible impact on the clearance of the three PRIs. Generally, these results demonstrated that diafiltration provides effective clearance for positively or neutrally charged LMW PRIs, while clearance for negatively charged and hydrophobic PRIs can be less effective. However, additional clearance can be achieved by extending diafiltration.

Overall, Protein A chromatography and cation exchange chromatography generally can provide greater than 1,000‐ and 100‐fold clearance for the PRIs, respectively. A typical 6‐diavolume diafiltration process can provide greater than 100‐fold clearance for positively and neutrally charged PRIs, while clearance of negatively charged or hydrophobic PRIs was impacted, but desired clearance can be achieved by extending the diafiltration process. For example, to get a 100‐fold clearance for EDTA and Tropolone, it would take about 5.7 and 8.2 DV, respectively.

### 3.10 Testing strategy and process development with leveraging clearance potentials

It is obvious that the remaining level of LMW PRIs should be the lowest at the end of the downstream process compared to any other intermediate steps (Figure 6a). When knowledge for the downstream process capability is limited, to account for the full clearance of the entire downstream, testing point for the PRIs can be set at end of the downstream process, usually at ultrafiltration/diafiltration pool (i.e., Point 3). Drug substance is usually not preferred because excipients in the formulation may interfere with analytical assays. Moreover, testing drug substance blurs the line between in‐process testing and release testing, leading to a misconception that the PRI is part of the release testing. On the other hand ultrafiltration/diafiltration products typically have high protein concentration, which often negatively impacts the analytical testing for PRIs. Sample dilution minimizes the impacts but also compromises higher assay sensitivity needed for demonstrating clearance. Therefore, UFDF and DS are not preferred test points for PRI risk assessment.

As clearance knowledge for the downstream process is gained, the testing strategy can be modified to simplify assay development and qualification. For example, for LMW PRIs introduced in the upstream process and during harvest of cell culture fluids, knowing that the PRIs can be effectively removed by Protein A chromatography can allow for the testing point to the Protein A elution pool (Point 1). In the platform process paradigm, the composition of the Protein A elution pool is likely similar for different mAbs, and therefore assay qualification may be leveraged across projects. At the same time, the clearance data from different mAbs and different wash buffers helps determine the true clearance capability of Protein A chromatography. Similarly, for downstream PRIs introduced prior to the cation exchange chromatography step, the testing point can be set to the cation exchange pool (Point 2).

Process development should leverage clearance capability and can benefit from established clearance knowledge.^4^ For example, detergent (such as Triton X‐100) can be used as an alternate to low pH for virus inactivation. As demonstrated in our study, Triton X‐100 was not removed well in bind‐and‐elute cation exchange chromatography, while its removal was excellent in Protein A chromatography. The removal of Triton X‐100 is similar to the clearance of detergent LDAO (lauryldimethylamine N‐oxide) on Protein A chromatography reported by Conley et al.^35^ Accordingly, virus inactivation using Triton X‐100 or other detergents is often carried out ahead of the Protein A chromatography step to maximize clearance and minimize safety risks. For mAbs entering late stage development, since the clearance capability for PRIs of the downstream process has been established with the historical data, maximum usage concentrations for PRIs can be defined based on cumulative clearance data. On one hand, process benefit from these PRIs can be maximized when greater usage amount brings in more process benefits; on the other hand, an effective control strategy is in place for the PRIs because of historical knowledge. Routine testing for some PRIs could be eliminated after robust removal has been supported by sufficient clearance data. Similarly, routine testing for some PRIs in mAbs that are manufactured by platform process can be reduced or even eliminated when robust clearance for the PRIs has been demonstrated.^4^

## 4 SUMMARY

The intent of this report was to bridge remaining gaps in the safety risk assessment for LMW PRIs in mAb drug substance/drug product and outline a systematic risk management process. We discussed categorization of PRIs according to their toxicological concerns and the corresponding risk assessment approaches that were proposed in recent publications.^4^, ^17^, ^23^ An integration of the safety risk management processes with CMC activities helps achieve effective risk management and potentially shorten development timelines. Through analysis of historical data from 28 mAbs and clearance data from systematic spike/clearance studies, we demonstrated that the typical mAb downstream process has the potential to provide robust clearance for all LMW PRIs. In‐process testing for many PRIs introduced in the upstream and harvest processes can be eliminated because of the robust removal demonstrated downstream. Based on our data, we propose that similar mAb downstream processes should have similar PRI clearance capability.

In terms of reasonably reducing testing burden, removal of all in‐process testing for PRIs and assuming good clearance certainly poses significant risks. Based on the results presented in this work, assuming no process clearance is highly conservative, and assuming some degree of process clearance for typical mAb downstream process has scientific basis and is reasonable. Our studies showed that Protein A chromatography and cation exchange chromatography (operated in bind‐elute mode) had greater than 1,000‐ and 100‐fold clearance, respectively. The typical diafiltration process is also capable of removing LMW PRIs, generally more than 100‐fold but the clearance potential can be affected by PRI chemical properties such as charge and hydrophobicity as demonstrated in this study and several recent reports.^24^, ^34^ The downstream clearance potential must be considered in the safety risk assessment to avoid any unnecessary testing. In the absence of clearance data, the initial risk assessment (Figures 2 and 3) based on the worst‐case assumption that there is no clearance during downstream processing is likely to lead to some testing. However, the clearance data presented here suggests that it is quite reasonable to assume some conservative level of clearance, which can help reduce the testing burden. For the overall process, a minimum clearance of 5,000‐fold can be assumed for mAb purification processes, with 100‐fold clearance from the Protein A chromatography step, 10‐fold clearance from the cation exchange chromatography (in bind‐elute mode), and fivefold clearance from the diafiltration process. With this assumption, an additional assessment taking into account a minimum process clearance can be added to the decision tree in Figure 2. After accumulating sufficient clearance data, testing for some LMW PRIs may be avoided for mAbs using the platform process. The gained clearance potential of each unit operation also facilitates the process development for a new mAb. It is noteworthy that clearance of dextran sulfate and Triton X‐100 by bind‐elute cation exchange chromatography was significantly lower than the clearance by Protein A chromatography. The poor clearance of dextran sulfate may be explained by the potential electrostatic interactions between dextran sulfate and the resins or bound mAbs. The mechanism for the retention of Triton X‐100 by the cation exchange chromatography would need further studies. Furthermore, we found that the properties (such as charge and hydrophobicity) of protein and/or PRI could impact the clearance during tangential flow filtration through potential weak interact rations between PRI and proteins. These undesired interaction led to lower sieving coefficients for several commonly seen PRIs. The sieving coefficients obtained in our study for the commonly seen LMW PRIs can be used to guide diafiltration development to achieve desired clearance. Taken together, this report establishes an effective safety risk management and rational design of robust downstream process for LMW PRIs.

## AUTHOR CONTRIBUTIONS

**Haibin Luo:** Conceptualization; data curation; formal analysis; funding acquisition; investigation; methodology; project administration; resources; software; supervision; validation; visualization; writing‐original draft; writing‐review and editing. **Yuling Li:** Conceptualization; formal analysis; funding acquisition; investigation; methodology; project administration; resources; supervision; validation; visualization; writing‐original draft; writing‐review and editing. **David Robbins:** Conceptualization; data curation; formal analysis; investigation; resources; validation; writing‐original draft; writing‐review and editing. **Sheau‐Chiann Wang:** Conceptualization; data curation; formal analysis; funding acquisition; investigation; methodology; project administration; resources; software; supervision; validation; visualization; writing‐original draft; writing‐review and editing. **Guoling Xi:** Formal analysis; investigation; methodology; resources; validation; writing‐original draft; writing‐review and editing. **Matthew Cox:** Investigation; resources. **Simone Nicholson:** Conceptualization; formal analysis; investigation; resources; validation; writing‐original draft; writing‐review and editing. **Chenghong Wei:** Conceptualization; formal analysis; investigation; resources; writing‐original draft; writing‐review and editing. **Timothy Pabst:** Investigation; resources; supervision; writing‐original draft; writing‐review and editing. **William Wang:** Conceptualization; formal analysis; funding acquisition; investigation; methodology; project administration; resources; supervision; visualization; writing‐original draft; writing‐review and editing.

5

### PEER REVIEW

The peer review history for this article is available at https://publons.com/publon/10.1002/btpr.3119.

## References

[1] ICHQ6B . Test procedures and acceptance criteria for biotechnological/biologcial products. 1999.

[2] Challenger CA. Expectations for residual impurity analysis continue to rise: more complex biologic samples must be evaluated to ever high levels of specificity and sensitivity. BioPharm Int. 2018;31(8):20‐25.

[3] Parasuraman S. Toxicological screening. J Pharmacol Pharmacother. 2011;2(2):74‐79. 21772764 10.4103/0976-500X.81895 PMC3127354

[4] Zhao X, Lin H, Qui J. Reagent clearance capability of protein a chromatography: a platform strategy for elimination of process reagent clearance testing. Bioprocess Int. 2015;13(5):8.

[5] ICH Q3A(R2) . Impurities in new drug substances. 2006.

[6] Group, C. B. W . A‐Mab: a case study in bioprocess development. 2009.

[7] Group, A. P. W . Quality by design for biotechnology products. 2013.

[8] Siew A. Impurity testing of biologic drug products. BioPharm Int. 2018;31(2):5.

[9] Teasdale A, Elder D, Nims RW. ICH Quality Guidelines: *An Implementation Guide (1st edition)*. New York: John Wiley & Sons, Inc., 2018.

[10] Wang X, Hunter AK, Mozier NM. Host cell proteins in biologics development: identification, quantitation and risk assessment. Biotechnol Bioeng. 2009;103(3):446‐458. 19388135 10.1002/bit.22304

[11] Bracewell DG, Francis R, Smales CM. The future of host cell protein (HCP) identification during process development and manufacturing linked to a risk‐based management for their control. Biotechnol Bioeng. 2015;112(9):1727‐1737. 25998019 10.1002/bit.25628 PMC4973824

[12] Hogwood CE, Bracewell DG, Smales CM. Host cell protein dynamics in recombinant CHO cells: impacts from harvest to purification and beyond. Bioengineered. 2013;4(5):288‐291. 23328085 10.4161/bioe.23382 PMC3813528

[13] Wang W, Ignatius AA, Thakkar SV. Impact of residual impurities and contaminants on protein stability. J Pharm Sci. 2014;103(5):1315‐1330. 24623189 10.1002/jps.23931

[14] Yang H. Establishing acceptable limits of residual DNA. PDA J Pharm Sci Technol. 2013;67(2):155‐163. 23569076 10.5731/pdajpst.2013.00910

[15] Yang H, Zhang J. A Bayesian approach to residual host cell DNA safety assessment. PDA J Pharm Sci Technol. 2016;70(2):157‐162. 26797975 10.5731/pdajpst.2015.006296

[16] Izhaky D, Pecht I. What else can the immune system recognize? Proc Natl Acad Sci U S A. 1998;95(20):11509‐11510. 9751695 10.1073/pnas.95.20.11509 PMC33900

[17] ICH Q3C (R6) , Impurities: Guideline For Residual Solvents. 2016.

[18] ICHQ3D(R1) . Guideline for Elemental Impurities. 2019.

[19] ICH Q9 , Quality risk management. 2005.

[20] ICH M7(R2) , Assessment and control of DNA reactive (mutagenic) impurities in pharmaceuticals to limit potential carcinogenic risk. 2018.

[21] Geigert J. Complex process‐related impurity profiles. In: Geigert, ed. The Challenge of CMC Regulatory Compliance for Biopharmaceuticals. Cham, Switzerland: J Springer; 2013;231‐260.

[22] Qiu J, Li K, Miller K, Raghani A. Risk‐based strategy to determine testing requirement for the removal of residual process reagents as process‐related impurities in bioprocesses. PDA J Pharm Sci Technol. 2015;69(3):334‐345. 26048741 10.5731/pdajpst.2015.01056

[23] Schenerman MA, Axley MJ, Oliver CN, Ram K, Wasserman GF. Using a risk assessment process to determine criticality of product quality attributes. In: Rathore AS, Mhatre R, eds. Quality by Design for Biopharmaceuticals: Principles and Case Studies. New Jersey: John Wiley & Sons, Inc.; 2008.

[24] Harinarayan C, Skidmore K, Kao Y, Zydney AL, van Reis R. Small molecule clearance in ultrafiltration/diafiltration in relation to protein interactions: Study of citrate binding to a Fab. Biotechnol Bioeng. 2009;102(6):1718‐1722. 19132743 10.1002/bit.22196

[25] Bradley EL, Speck DR, Read WA, Castle L. Method of test and survey of caprolactam migration into foods packaged in nylon‐6. Food Addit Contam. 2004;21(12):1179‐1185. 15799563 10.1080/02652030400023093

[26] Wishart DS, Feunang YD, Marcu A, et al. HMDB 4.0: the human metabolome database for 2018. Nucleic Acids Res. 2018;46(D1):D608‐D617. 29140435 10.1093/nar/gkx1089 PMC5753273

[27] Wishart DS, Tzur D, Knox C, et al. HMDB: the human metabolome database. Nucleic Acids Res. 2007;35(Database issue):D521‐D526. 17202168 10.1093/nar/gkl923 PMC1899095

[28] Weidolf L, Andersson T, Bercu JP, et al. Qualification of impurities based on metabolite data. Regul Toxicol Pharmacol. 2019;110:104524. 31734179 10.1016/j.yrtph.2019.104524

[29] FDA Guidedance (FDA‐2020‐D‐1530): Control of nitrosamine impurities in human drugs. 2020.

[30] Shukla AA, Wolfe LS, Mostafa SS, Norman C. Evolving trends in mAb production processes. Bioeng Transl Med. 2017;2(1):58‐69. 29313024 10.1002/btm2.10061 PMC5689530

[31] Pachekrepapol U, Horne DS, Lucey JA. Effect of dextran and dextran sulfate on the structural and rheological properties of model acid milk gels. J Dairy Sci. 2015;98(5):2843‐2852. 25747831 10.3168/jds.2014-8660

[32] Goddard ED, Turro NJ, Kuo PL, Ananthapadmanabhan KP. Fluorescence probes for critical micelle concentration determination. Langmuir. 1985;1(3):352‐355. 21370917 10.1021/la00063a015

[33] Miao F, Velayudhan A, DiBella E, et al. Theoretical analysis of excipient concentrations during the final ultrafiltration/diafiltration step of therapeutic antibody. Biotechnol Prog. 2009;25(4):964‐972. 19569193 10.1002/btpr.168

[34] Shao J, Zydney AL. Retention of small charged impurities during ultrafiltration. Biotechnol Bioeng. 2004;87(1):7‐13. 15211483 10.1002/bit.20009

[35] Conley L, Tao Y, Henry A, et al. Evaluation of eco‐friendly zwitterionic detergents for enveloped virus inactivation. Biotechnol Bioeng. 2017;114(4):813‐820. 27800626 10.1002/bit.26209

[36] Bannai S. [Use of 2‐mercaptoethanol in cell culture]. *Hum Cell* 1992;5(3):292‐297. 1467329

[37] Chaderjian WB, Chin ET, Harris RJ, Etcheverry TM. Effect of copper sulfate on performance of a serum‐free CHO cell culture process and the level of free thiol in the recombinant antibody expressed. *Biotechnol Prog*. 2005;21(2):550‐553. 10.1021/bp0497029 15801797

[38] Magarian N, Lee K, Nagpal K, Skidmore K, Mahajan E. Clearance of extractables and leachables from single‐use technologies via ultrafiltration/diafiltration operations. *Biotechnol Prog*. 2016;32(3):718‐724. 10.1002/btpr.2277 27071939

[39] Hyoung Park J, Sin Lim M, Rang Woo J, Won Kim J, Min Lee G. The molecular weight and concentration of dextran sulfate affect cell growth and antibody production in CHO cell cultures. *Biotechnol Prog*. 2016;32(5): 1113‐1122. 10.1002/btpr.2287 27114230

[40] Trexler‐Schmidt M, Sargis S, Chiu J, Sze‐Khoo S, Mun M, Kao YH, Laird MW. Identification and prevention of antibody disulfide bond reduction during cell culture manufacturing. *Biotechnol Bioeng*. 2010;106(3):452‐461. 10.1002/bit.22699 20178122

[41] Jiang C, Scherfner S, Dick LW, Jr. Mahon D, Qiu D, Cheng KC, Shukla AA. Demonstrating beta‐glucan and yeast peptide clearance in biopharmaceutical downstream processes. *Biotechnol Prog*. 2011;27(2):442‐450. 10.1002/btpr.568 21365784

[42] Jeon M, Lim JB, Lee GM. Development of a serum‐free medium for in vitro expansion of human cytotoxic T lymphocytes using a statistical design. *BMC Biotechnol*. 2010;10:70. 10.1186/1472-6750-10-70 PMC2954940 20854694

[43] Feary M, Racher AJ, Young RJ, Smales CM. Methionine sulfoximine supplementation enhances productivity in GS‐CHOK1SV cell lines through glutathione biosynthesis. *Biotechnol Prog*. 2017;33(1):17‐25. 10.1002/btpr.2372 27689785

[44] Kumar V, Sharma VK, Kalonia DS. Effect of polyols on polyethylene glycol (PEG)‐induced precipitation of proteins: Impact on solubility, stability and conformation. *Int J Pharm*. 2009;366(1‐2):38‐43. 10.1016/j.ijpharm.2008.08.037 18809481

[45] Tharmalingam T, Goudar CT. Evaluating the impact of high Pluronic(R) F68 concentrations on antibody producing CHO cell lines. *Biotechnol Bioeng*. 2015;112(4):832‐837. 10.1002/bit.25491 25384465

[46] Wang S, Wu G, Zhang X, Tian Z, Zhang N, Hu T, Dai W, Qian F. Stabilizing two IgG1 monoclonal antibodies by surfactants: Balance between aggregation prevention and structure perturbation. *Eur J Pharm Biopharm*. 2017;114:263‐277. 10.1016/j.ejpb.2017.01.025 28189625

[47] Velugula‐Yellela SR, Williams A, Trunfio N, Hsu CJ, Chavez B, Yoon S, Agarabi C. Impact of media and antifoam selection on monoclonal antibody production and quality using a high throughput micro‐bioreactor system. *Biotechnol Prog*. 2018;34(1):262‐270. 10.1002/btpr.2575 PMC5821576 29086492

[48] Roberts PL. Virus inactivation by solvent/detergent treatment using Triton X‐100 in a high purity factor VIII. *Biologicals* 2008;36(5):330‐335. 10.1016/j.biologicals.2008.06.002 18674928

[49] Zhang J, Robinson D. Development of Animal‐free, Protein‐Free and Chemically‐Defined Media for NS0 Cell Culture. *Cytotechnology* 2005;48(1‐3):59‐74. 10.1007/s10616-005-3563-z PMC3449720 19003032

